# Unsupervised anomaly detection for earthquake detection on Korea high-speed trains using autoencoder-based deep learning models

**DOI:** 10.1038/s41598-024-51354-7

**Published:** 2024-01-05

**Authors:** Jeonguk Seo, Yunu Kim, Jisung Ha, Dongyoup Kwak, Minsam Ko, Mintaek Yoo

**Affiliations:** 1 Department of Applied Artificial Intelligence, Hanyang University ERICA, Ansan-si, South Korea; 2 Department of Applied Artificial Intelligence (Major in Bio Artificial Intelligence), Hanyang University ERICA, Ansan-si, South Korea; 3 Department of Civil and Environmental Engineering, Hanyang University ERICA, Ansan-si, South Korea; 4 Department of Civil and Environmental Engineering, Gachon University, Seongnam-si, South Korea

**Keywords:** Natural hazards, Engineering

## Abstract

We propose a method for detecting earthquakes for high-speed trains based on unsupervised anomaly-detection techniques. In particular, we utilized autoencoder-based deep learning models for unsupervised learning using only normal training vibration data. Datasets were generated from South Korean high-speed train data, and seismic data were measured using seismometers nationwide. The proposed method is compared with the conventional Short Time Average over Long Time Average (STA/LTA) model, considering earthquake detection capabilities, focusing on a Peak Ground Acceleration (PGA) threshold of 0.07, a criterion for track derailment. The results show that the proposed model exhibit improved earthquake detection capabilities than STA/LTA for PGA of 0.07 or higher. Furthermore, the proposed model reduced false earthquake detections under normal operating conditions and accurately identified normal states. In contrast, the STA/LTA method demonstrated a high rate of false earthquake detection under normal operating conditions, underscoring its propensity for inaccurate detection in many instances. The proposed approach shows promising performance even in situations with limited seismic data and offers a viable solution for earthquake detection in regions with relatively few seismic events.

## Introduction

Historically, the Korean Peninsula has been considered safe from earthquakes. However, awareness of seismic events has increased with the 2016 Gyeongju earthquake^1^ and 2017 Pohang earthquake^2^. Earthquakes incur social costs owing to structural damage and collapse, as well as secondary damages, such as vehicle overturning owing to structural failure.

Hence, it is crucial to establish an earthquake early warning system while ensuring the seismic resilience of structures. Of particular concern are railway facilities, such as railway bridge collapse, embankment settlement, tunnel lining failure, bridge pier lateral displacement, and landslides, which are significant social infrastructures that can suffer structural damage ^3–11^. Such damage can lead to train derailment and collisions during operation, resulting in significant human and property losses.

An earthquake detection and warning system capable of promptly slowing or halting trains in the event of an earthquake is required to mitigate these risks. High-speed train operations in South Korea commenced in 2004, with an initial maximum speed of 300 km/h, connecting Seoul and Busan. Subsequently, in 2007, the country embarked on a next-generation high-speed railway technology development project that has completed its design phase and is currently in production. The project's flagship achievement, the HEMU 400x (Next Generation High-Speed train (HEMU), boasts a remarkable maximum speed of 430 km/h. It is projected that this new train model will increase passenger transportation capacity by approximately 16% compared to Korean high-speed railway-Sancheon (KTX-Sancheon).

Development efforts go beyond speed enhancement. They also focus on integrating cutting-edge electric/control, information communication, and IT technologies with high-speed trains. The goal is to create a sophisticated, interconnected system that enhances overall efficiency and user experience.

High-speed railway systems primarily consist of critical structures including earthworks, tunnels, and bridges. However, recent seismic activity trends on the Korean Peninsula have raised concerns about the safety of these structures. Earthquake occurrence rates have rapidly increased since 2015. The Gyeongju earthquake, considered the most significant seismic event since earthquake observation began, exceeded the seismic design standards for specific vibration frequency segments within the acceleration response spectrum. Similarly, the Pohang earthquake caused severe damage to buildings and equipment.

Given these seismic challenges, it has become increasingly essential to implement advanced earthquake detection and warning systems that can safeguard high-speed train operations and protect passengers and infrastructure. Early warning systems are essential for railway systems to halt train operations when anticipating earthquake damage. The current alarm system for high-speed railways in Korea notifies engineers during emergencies to decide on train operations^12,13^.

**Figure 1:**
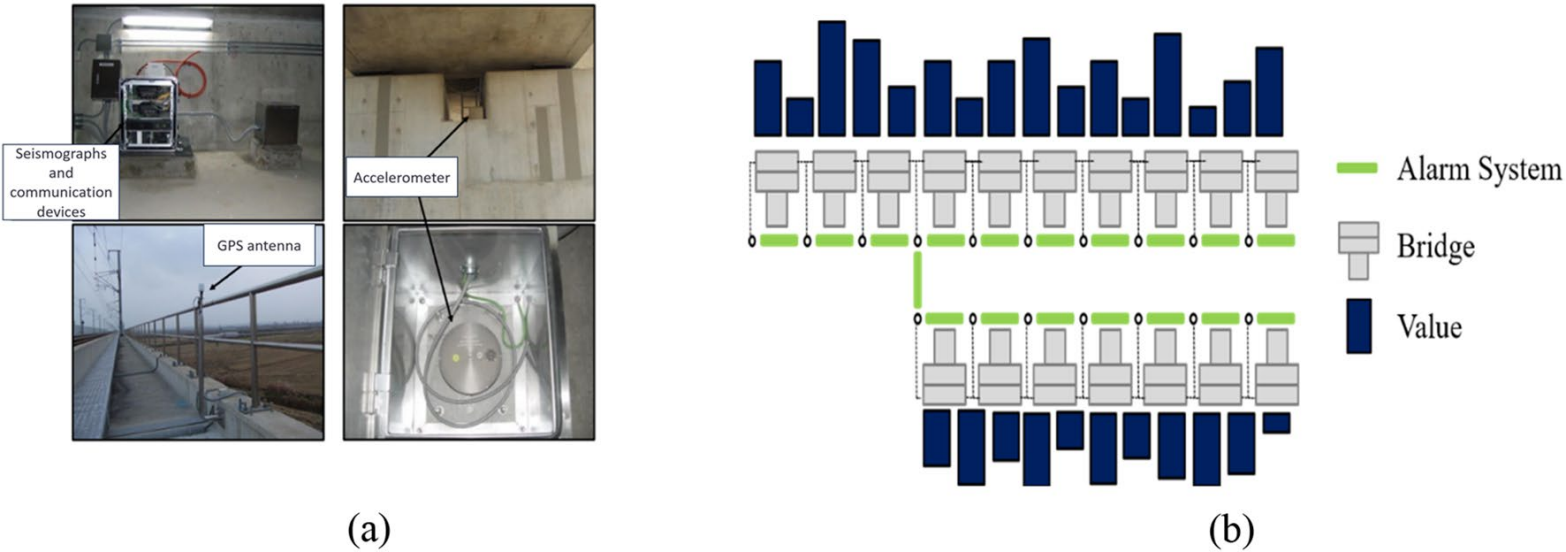
(**a**) Installed sensor on bridge (**b**). Real-time monitoring screen of earthquake early warning system.

**Figure 2:**
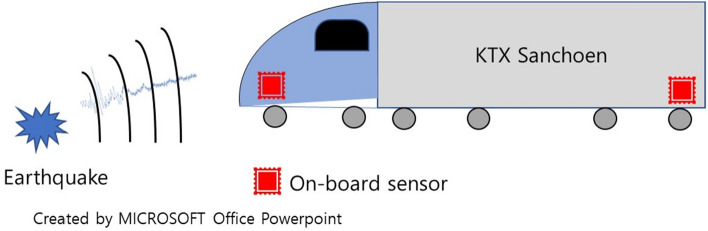
Train with On-board Detected Earthquake.

Earthquake acceleration sensors are strategically installed along the Korean high-speed railway (KTX) at intervals of 20–30 km, particularly in the main pier and tunnel areas. Additionally, seismic drilling acceleration sensors are placed in a free field away from the track and pier structures for real-time monitoring of the maximum acceleration values. This comprehensive seismic detection system enables the efficient operation of high-speed railways under seismic conditions. Figure 1 shows the earthquake warning system currently in operation in Korea. In order to detect the earthquake occurrence, total 48 seismometers installed in railway bridge pier every 12 km along the KTX Gyeongbu line as shown in Fig. 1a. The acceleration values of seismometers are shown in the real-time monitoring system for the earthquake early warning as shown in Fig. 2a, and allows constant checks of the vibration status of each railway section. In addition, automatic alerts are triggered when the vibration exceeds a certain threshold.

However, existing earthquake warning systems require a considerable time to stop a train after an earthquake. Moreover, the lack of earthquake acceleration sensors in certain areas along railways makes it difficult to confirm whether an earthquake has occurred when a train passes through such sections.

Thus, there is an urgent need to develop a novel earthquake warning system to address the high costs associated with the installation and maintenance of earthquake acceleration sensors across all railway sections. Recently, earthquake warning network technologies utilizing smart devices and the Internet have gained significant attention. For example, the Quake Catcher Network developed by the Southern California Earthquake Center and the Incorporated Research Institutions for Seismology in the US, has made strides in this field^14^. Caltech in the US has also made advancements in a Community Seismic Network, an earthquake observation network employing USB seismometers^15^. Furthermore, MyShake application, which utilizes smartphone sensors for earthquake observations, boasts of over 200,000 users. Studies have shown that MyShake can detect and issue earthquake warnings faster and more accurately than ShakeAlert, a US earthquake-warning system^16^.

However, despite these advances in earthquake warning networks, there remains a gap in the development of networks specifically designed for railways that utilize onboard sensors in train vehicles. Detecting earthquakes in train vehicles is more challenging than identifying earthquakes in building structures. In building structures, detecting earthquake vibrations is relatively straightforward due to the occurrence of small vibrations even in the absence of actual earthquakes. In contrast, train vehicles experience significant vibrations during normal train operations, necessitating additional analytical models to distinguish the unique vibration characteristics associated with actual seismic events.

The conventional approach to earthquake detection involves using the short-term average/long-term average (STA/LTA) technique, which calculates the ratio of a signal's short and long time windows^17^. However, this method often leads to false earthquake identification. Hence, there has been growing interest in utilizing deep learning techniques for earthquake detection, which have proven effective even for small earthquakes. The two primary methods used for implementing deep learning in this context are supervised and unsupervised learning. In supervised learning, data are labeled as either earthquake or nonearthquake, whereas in unsupervised learning, such labels are not required. Various studies have explored earthquake detection using supervised learning methods^18,19^; however, these approaches require numerous labeled earthquake samples, which can be difficult to acquire, particularly for trains. Therefore, an unsupervised learning approach that can learn without labeled data is essential. However, despite the potential of unsupervised learning, there are limited instances of its application in earthquake detection on trains. To address these challenges, this study proposes an unsupervised deep learning method for earthquake detection on trains.

Unsupervised anomaly detection is considered one of the leading methodologies in unsupervised learning. This enables the identification of anomalies in the data without requiring labeled anomalous instances^20–22^. Anomaly detection aims to identify abnormal data points and is crucial for analyzing seismic data because of the scarcity of abnormal instances and their labels compared with normal data. Therefore, unsupervised learning-based anomaly detection methods rely solely on normal data to train deep neural network models, leading to superior performance compared to traditional methods in anomaly detection.

In this study, we aimed to develop an earthquake warning system using an onboard sensor capable of measuring earthquakes on trains, thus replacing earthquake acceleration sensors in railroad facilities. Because the onboard acceleration sensor measures both train and earthquake-induced vibrations, it is essential to distinguish between them. Therefore, anomaly detection model which can distinguishes between normal train vibration and another anomaly vibration was developed, and it was verified using seismic vibration data. The seismic vibration data used for verification is secondary waves, which can cause directly damage to train operations. Figure 2 shows the setup with a low-cost MEMS sensor installed inside the train to detect earthquake signals and alert train operators. The proposed warning system estimates the probability of potential damage based on the amplitude of the earthquake signal recorded by the MEMS sensor. An unsupervised anomaly-detection method was proposed for detecting earthquakes while the train was in motion, eliminating the need for labeled earthquake data during training.

## Related works

### Train vibration

**Figure 3:**
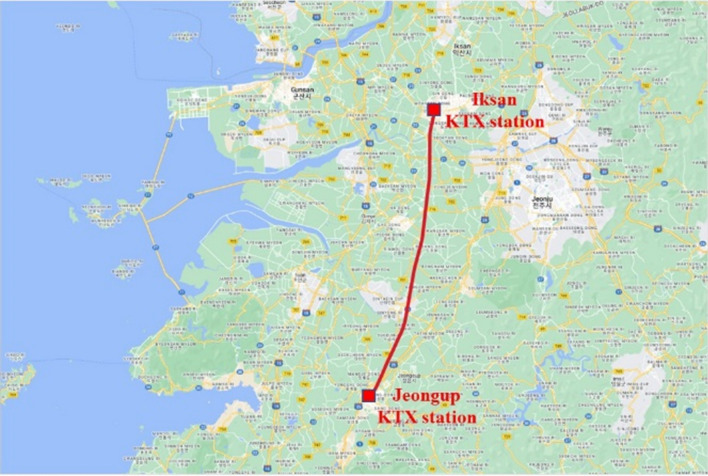
Vibration data measured route (KTX Iksan–KTX Jeoungyeup), Map data: Google.

**Figure 4:**
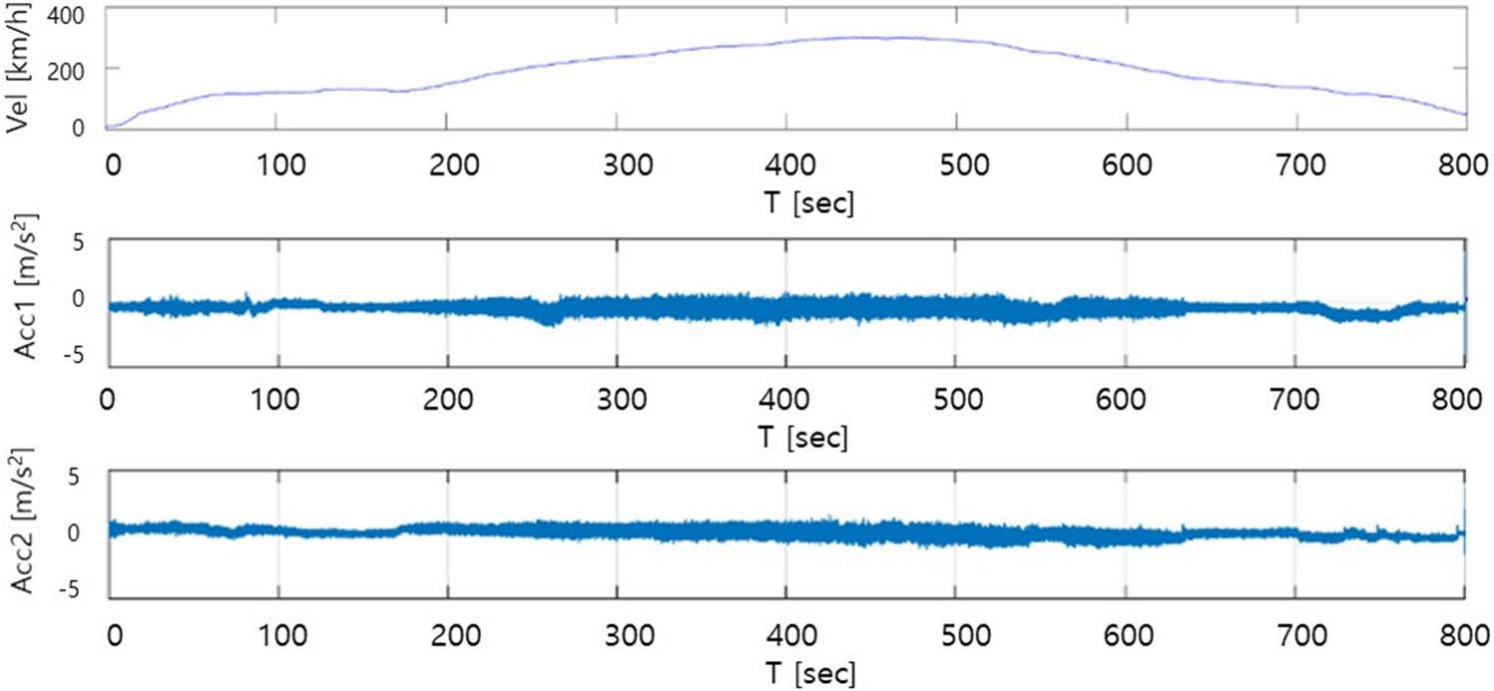
Example of KTX train vehicle velocity and vibration.

As illustrated in Fig. 3, train acceleration measurements were conducted on the segment between KTX Iksan and KTX Jeongeup Stations. This section is part of the KTX Honam High-Speed Railway and comprises mostly straight tracks, minimizing the impact of curved rails on train vibration. The train accelerates to its maximum speed of 300 km/h and then decelerates to a halt, making it suitable for capturing train acceleration data at various speed ranges. A custom-designed sensor unit combining a Raspberry Pi and an IMU sensor was employed for data collection. The sampling rates of the Raspberry Pi and IMU sensors were set to 0.01 s. Acceleration measurements were performed along the three axes (x, y, and z) for approximately 800 s. To assess accuracy, sensors were installed at the front and rear of the train vehicle. Figure 4 shows the time history of the lateral acceleration due to the KTX train vibration, measured using the Raspberry Pi and IMU sensors.

Jae Sang Moon and Mintaek Yoo (2020)^23^, conducted an extensive frequency analysis of the Korean high-speed train vibration data, used in this paper. The analysis was carried out using Short-Time Fourier Transform (STFT) and Power Spectral Density (PSD) techniques. The authors examined the vibration data in both the longitudinal and transverse directions, employing sampling rates of 4000 Hz and 500 Hz, respectively. Furthermore, this study involved the analysis of seismic data, which can contribute as extraneous noise affecting the train. The analysis of seismic data was carried out using STFT and PSD techniques. Subsequently, the results of the frequency analysis of the train were compared with the analysis of seismic data.

### Seismic vibration

**Figure 5:**
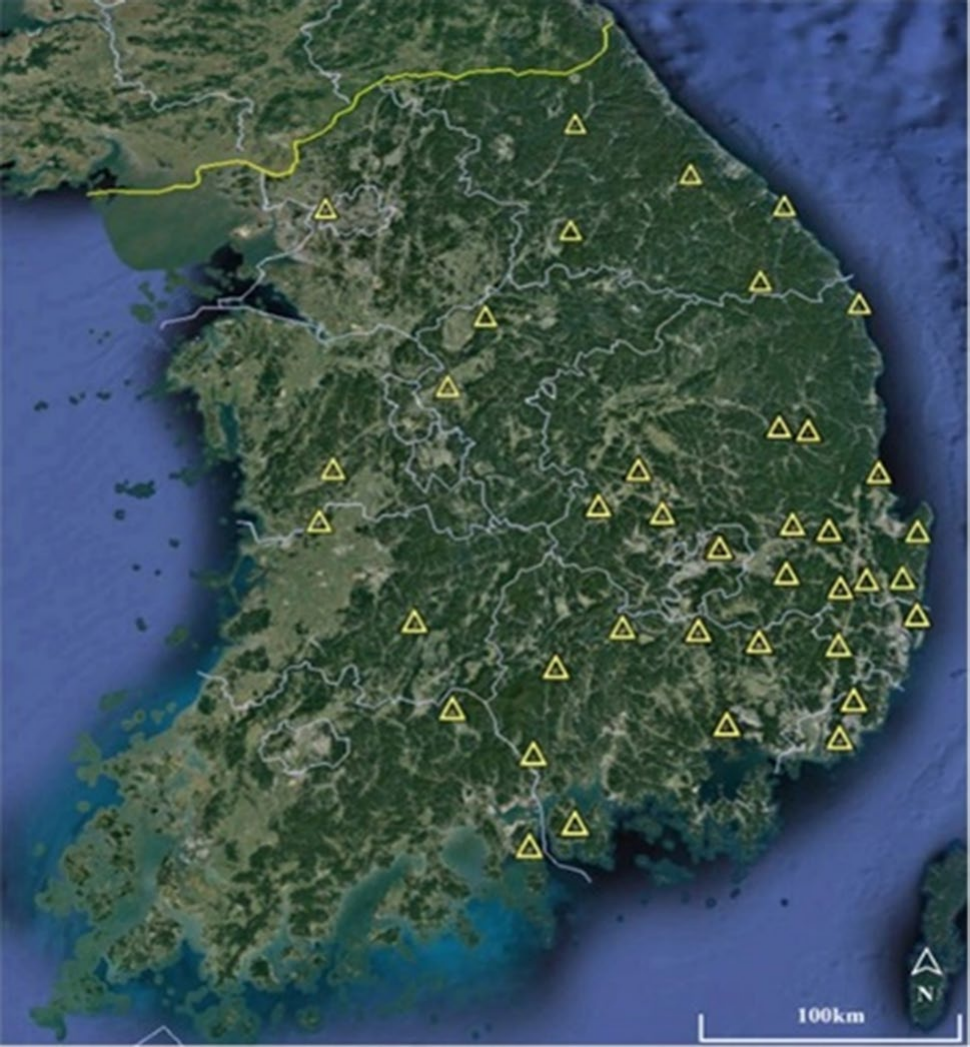
Earthquake measuring stations in Korea, Map data: Google.

The seismic vibration dataset was compiled using earthquake data with magnitude of 2.0 or higher, in the Korean Peninsula between 2007 and 2017. Earthquake data were acquired from an earthquake measuring station, as shown in Fig. 5. The dataset encompasses earthquakes with magnitudes of 5 or greater in Korea. Additionally, supplementary earthquake data were randomly selected from the measured earthquake records to augment the dataset.

**Figure 6:**
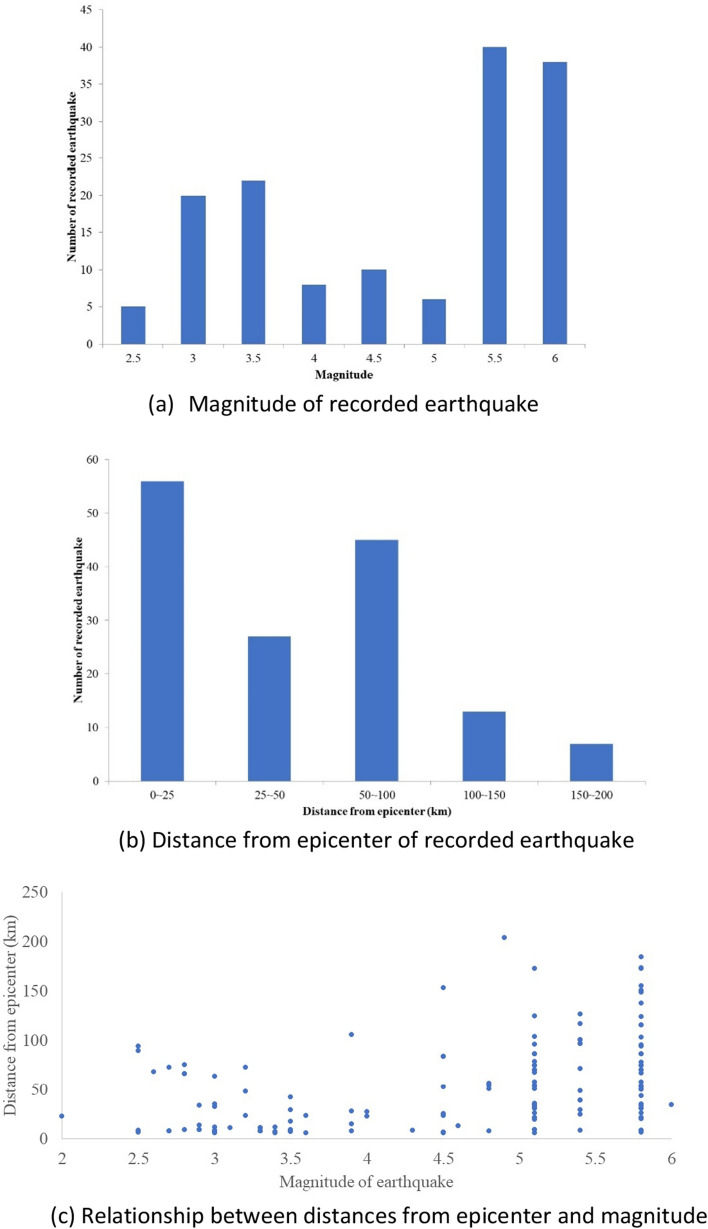
Magnitude of earthquake vibration.

**Figure 7:**
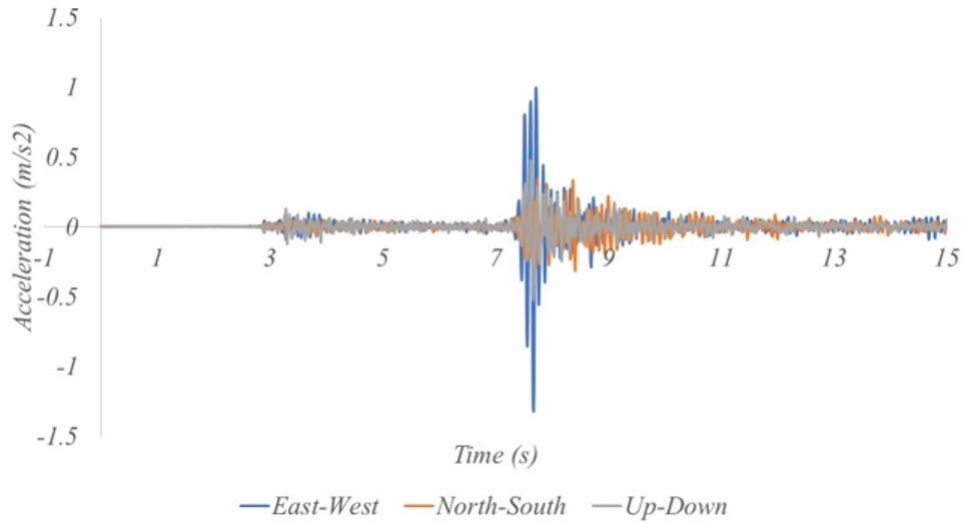
Example of earthquake vibration data (Magnitude of 5.8, Distance from epicenter of 52 km).

Total 149 earthquake datasets were constructed for this research. Fig. 6 illustrates the distribution of earthquake data according to magnitude, distance from epicenter and relationship between two values. The measurable distance of an earthquake at an observation station varies depending on the magnitude of the earthquake, however it is confirmed that it can measure up to more than 50 km for magnitude 3, over 100 km for magnitude 4, and over 150 km for magnitude 5 or higher. Therefore, it is possible to measure earthquakes up to the corresponding distances for each magnitude in an actual railway structure. In addition, Fig. 7 shows the time history of the earthquake vibration data recorded in the three directions obtained from the earthquake measuring station.

In addition, the seismic acceleration could measure in the train vehicle transferred depending on the structural response and the railway-vehicle interaction response. Yoo et al. (2022)^24^ developed a transfer function that can calculate seismic acceleration that can be measured within a train by considering structural response and railway-vehicle interaction. In this study, transfer function developed previous research was applied to derive the final seismic acceleration to be used for anomaly detection model.

## Methods

### Traditional method

In the conventional earthquake detection field, various methods, such as Continuous Wavelet Transform, frequency domain analysis, and real-time satellite data analysis, have been employed. Among these approaches, the STA/LTA is the most widely adopted conventional technique for earthquake detection.

*STA/LTA*: STA/LTA algorithm is widely used for event detection in microseismic interpretations. It analyzes seismic noise by computing the ratio between the STA and LTA, providing an indication of instantaneous changes. The STA/LTA ratio is calculated using the following formula:

1 $$ {\text{STA}}\left( {\text{t}} \right) = \frac{1}{{\text{N}}}\mathop \sum \limits_{{{\text{i}} = {\text{t}} - {\text{N}}}}^{{\text{t}}} {\text{x}}_{{\text{i}}}^{2} $$

2 $$ {\text{LTA}}\left( {\text{t}} \right) = \frac{1}{{\text{M}}}\mathop \sum \limits_{{{\text{i}} = {\text{t}} - {\text{M}}}}^{{\text{t}}} {\text{x}}_{{\text{i}}}^{2} $$

3 $$ {\text{STA}}/{\text{LTA}}\left( {\text{t}} \right) = \frac{{{\text{STA}}\left( {\text{t}} \right)}}{{{\text{LTA}}\left( {\text{t}} \right)}} $$

These equations illustrate the concepts of Short-Term Average and Long-Term Average, where ${\text{N}}$ represents the number of data points over a short time period, ${\text{M}}$ represents the number of data points over an extended time period, and ${\text{x}}_{{\text{I}}}$ denotes the data point at time ${\text{t}}$.

where STA and LTA represent the numbers of samples in the time windows used to compute the short- and long-term averages, respectively. CF refers to the characteristic function. We used the energy measurement method (McEvilly & Majer, 1982)^26^ to quantify the amplitude levels of the seismic samples. In the STA/LTA algorithm, event detection is triggered when the calculated ratio exceeds a predefined threshold.

*Recursive STA/LTA*: Recursive Short-Term Average/Long-Term Average (STA/LTA) methodology entails real-time updating of STA and LTA calculations while employing a dynamic moving window in lieu of static time windows. Continuous updates of STA and LTA values are executed to ensure the integration of the latest data, facilitating the real-time detection of seismic signals. The Recursive STA/LTA method is computed analogously to data structures with temporal overlays or through the utilization of techniques akin to moving averages, fostering its efficacy in the real-time monitoring of seismic events.

### Deep neural network models

In this study, we present a novel approach for anomaly detection in train vibration and seismic data using unsupervised learning-based deep neural networks, namely, autoencoder (AE), convolutional autoencoder (Conv-AE), and long short-term memory autoencoder (LSTM-AE).

This method allows us to distinguish earthquakes (anomalies) by learning only with train vibration data (normal data). Among the unsupervised learning models, the AE has emerged as the most popular and representative choice for this purpose. We have employed not only the AE but also its derivative models, namely LSTM-AE and Conv-AE.

The AE has emerged as the most popular and representative choice for this purpose. We have employed not only the AE but also its derivative models, namely LSTM-AE and Conv-AE. These derivative models are particularly suited for the analysis of time series data, as they leverage the inherent ability of Long Short-Term Memory (LSTM) and Convolutional Neural Network (CNN) architectures to capture temporal context. LSTM-AE integrates LSTM into the Autoencoder framework, while Conv-AE adapts CNN to the same architecture. By using these representative unsupervised learning models, including AE, Conv-AE, and LSTM-AE, we have conducted anomaly detection on time series data.

These unsupervised learning models were trained solely on the training vibration data to capture normal patterns. They reconstructed the data during the training process, allowing them to learn the inherent structure of the training vibration data without relying on seismic labels.

After training, these models can predict the anomaly scores by measuring the dissimilarity between the reconstructed and test data. Abnormal test data tended to exhibit higher anomaly scores than normal test data. By setting a predefined threshold, unsupervised learning-based models can effectively identify abnormal data and flag instances with anomaly scores surpassing the threshold as anomalies. This approach enables the detection of seismic anomalies in training vibration data without the need for labeled seismic data during the training phase, making it highly applicable to scenarios in which acquiring such labeled data is challenging.

**Figure 8:**
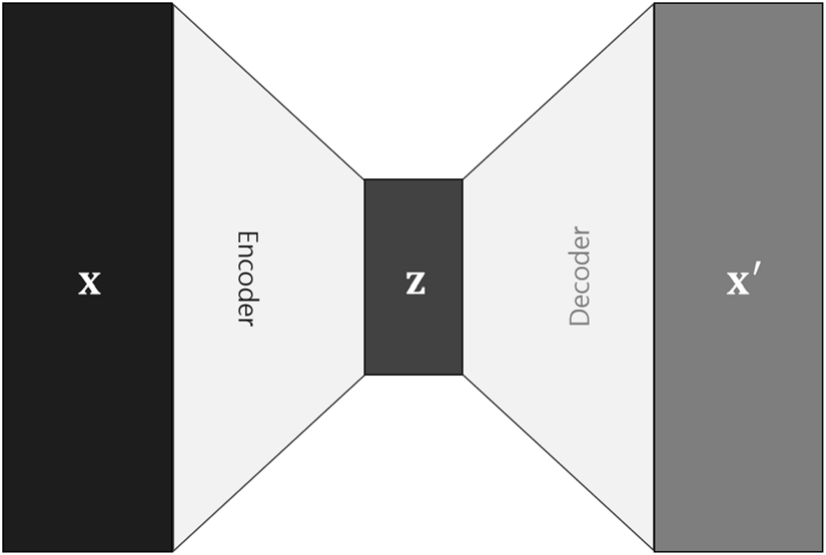
Autoencoder architecture. An autoencoder comprises an encoder and a decoder, with an intermediary latent vector capturing essential features of the input data. This latent vector serves as input to the decoder, generating output data that encapsulates the key features of the input. In certain instances, the encoder and decoder components of this autoencoder are adapted as Conv-AE or LSTM-AE by substituting them with CNN or LSTM.

*Autoencoder (AE)*: AE is a prevalent deep learning model utilized for data representation and generation and is commonly employed in unsupervised anomaly detection tasks. As illustrated in Fig. 8, Its architecture simplifies the input data by reducing its dimensionality and then reconstructs it back to its original form. (Hinton, Geoffrey E., and Salakhutdinov, Ruslan R, 2006)^25^ This process allows the model to extract essential features or core characteristics of the data by compressing its dimensions. Consequently, the autoencoder can generate new data with patterns similar to those of the original data based on the learned core characteristics. In this study, we utilized an autoencoder as an unsupervised learning-based model to detect anomalies in training vibration and seismic data. The AE is calculated using the following formula:

4 $$ {\mathbf{z}} = f_{{{\text{enc}}}} \left( {\mathbf{x}} \right) = {\upsigma }\left( {{\mathbf{W}}_{{{\text{enc}}}} {\mathbf{x}} + {\mathbf{b}}_{{{\text{enc}}}} } \right) $$

5 $$ {\mathbf{x^{\prime}}} = f_{{{\text{dec}}}} \left( {\mathbf{z}} \right) = {\upsigma }\left( {{\mathbf{W}}_{{{\text{dec}}}} {\mathbf{z}} + {\mathbf{b}}_{{{\text{dec}}}} } \right) $$

where ${\mathbf{x}}$ denotes the input data, ${\mathbf{x^{\prime}}}$ represents the reconstructed data, and ${\mathbf{z}}$ signifies the encoded feature vector. $f_{{{\text{enc}}}}$ stands for the encoder function, while $f_{{{\text{dec}}}}$ corresponds to the decoder function. The function $\sigma$ denotes the activation function. ${\mathbf{W}}_{{{\text{enc}}}}$ and ${\mathbf{W}}_{dec}$ refer to the weight matrices, whereas ${\mathbf{b}}_{{{\text{enc}}}}$ and ${\mathbf{b}}_{{{\text{dec}}}}$ represent the bias vectors.

The AE offer a straightforward and intuitive structure with encoder and decoder components, making them widely applicable and frequently employed models. Their simplicity facilitates easy implementation. However, one limitation is their inability to capture temporal context, which can pose challenges for effective processing of time series data.

*Convolutional autoencoder (Conv-AE)*: Conv-AE is an extension of the traditional autoencoder model in which the dense layer is substituted with a convolutional layer. This adaptation allowed the model to handle data with better temporal or spatial structures. The convolutional layer is adept at feature extraction while retaining the spatial structure of the data, making it particularly well-suited for datasets exhibiting such characteristics. In our research, we employed the Conv-AE as an unsupervised learning-based model to effectively detect anomalies in train vibration and seismic data, taking advantage of its ability to capture spatial patterns. The Conv-AE is calculated using the following formula:

6 $$ {\mathbf{z}} = f_{{{\text{enc}}}} \left( {\text{x}} \right) = {\upsigma }\left( {{\mathbf{W}}_{{{\text{enc}}}} {*}{\mathbf{x}} + {\mathbf{b}}_{{{\text{enc}}}} } \right) $$

7 $$ {\mathbf{x^{\prime}}} = f_{{{\text{dec}}}} \left( {\text{z}} \right) = {\upsigma }\left( {{\mathbf{W}}_{{{\text{dec}}}} {*}{\mathbf{z}} + {\mathbf{b}}_{{{\text{dec}}}} } \right) $$

where ${\mathbf{x}}$ represents an input data, and ${\mathbf{z}}$ denotes its corresponding latent representation. The encoder function $f_{{{\text{enc}}}}$ applies convolutions followed by activation functions to map ${\mathbf{x}}$ to ${\mathbf{z}}$. Similarly, the decoder function $f_{{{\text{dec}}}}$ employs convolutional transposed operations followed by activations to generate the reconstructed image ${\mathbf{x^{\prime}}}$ from ${\mathbf{z}}$**.**

8 $$ {\mathbf{W}}_{{{\text{enc}}}} {*}{\mathbf{x}} = \mathop \sum \limits_{m} \mathop \sum \limits_{n} {\mathbf{x}}\left( {i - m,j - n} \right) \cdot {\mathbf{W}}\left( {m,n} \right) $$

9 $$ {\mathbf{W}}_{{{\text{dec}}}} {*}{\mathbf{z}} = \mathop \sum \limits_{m} \mathop \sum \limits_{n} {\mathbf{x}}\left( {i + m,j + n} \right) \cdot {\mathbf{W}}\left( {m,n} \right) $$

In contrast to an AE, The Conv-AE distinguishes itself by employing convolution and deconvolution operations in both the encoding and decoding stages. Convolutions represent a fundamental operation that convolves the input data with a filter, primarily serving the purpose of generating feature maps. On the other hand, deconvolution, often referred to as the transposed convolution operation, acts as the inverse of convolution and is primarily employed for data upscaling and restoration. In the context of a Conv-AE, the decoder component utilizes inverse convolutions to reconstruct the original input. In the mathematical expressions, ${\mathbf{x}}$ denotes the input data, ${\mathbf{W}}$ represents the filter, ($i$, $j$) are the coordinates within the output feature map, and ($m$, $n$) are indices corresponding to the filter.

In the case of Conv-AE, that excel in handling 2D data, such as images, thanks to their capability to leverage spatial information through Convolutional Neural Networks (CNNs). Notably, time series data, although 1D in nature, can also be treated as having one-dimensional spatial characteristics. This enables Conv-AE to consider temporal context in time series data analysis. In such instances, configuring the model as a 1D CNN is essential for optimal performance.

*LSTM autoencoder (LSTM-AE)*: The LSTM-AE is a fusion of the LSTM and Autoencoder models. In this architecture, each layer comprises an LSTM layer, which enables the model to effectively extract features from sequential data. By incorporating an autoencoder structure, the LSTM-AE can capture the unique characteristics present in sequential data and generate new data based on the knowledge of these learned features. This combination empowers the LSTM-AE to excel in detecting anomalies in sequential data, such as train vibration and seismic data, by leveraging the strengths of both the LSTM and Autoencoder models. The LSTM-AE is calculated using the following formula:

10 $$ {\mathbf{z}}_{t} ,{\mathbf{h}}_{t} ,{\mathbf{c}}_{t} = {\text{LSTM}}_{{{\text{enc}}}} \left( {{\mathbf{x}}_{t} ,{\mathbf{h}}_{t - 1} ,{\mathbf{c}}_{t - 1} } \right) $$

11 $$ {\mathbf{x^{\prime}}}_{t} ,{\mathbf{h^{\prime}}}_{t} ,{\mathbf{c^{\prime}}}_{t} = {\text{LSTM}}_{{{\text{dec}}}} \left( {{\mathbf{z}}_{t} ,{\mathbf{h^{\prime}}}_{t - 1} ,{\mathbf{c^{\prime}}}_{t - 1} } \right) $$

where $t$ is time, ${\mathbf{x}}_{{\mathbf{t}}}$ represents the input data, ${\mathbf{x^{\prime}}}_{{\varvec{t}}}$ signifies the reconstructed data, and ${\mathbf{z}}_{{\varvec{t}}}$ denotes the encoded feature vector. The hidden state of the encoder LSTM is denoted as ${\mathbf{h}}_{{\varvec{t}}}$, and its cell state as ${\mathbf{c}}_{{\varvec{t}}}$. On the decoder side, ${\mathbf{h^{\prime}}}_{{\varvec{t}}}$ represents the hidden state of the decoder LSTM, while ${\mathbf{c^{\prime}}}_{{\varvec{t}}}$ indicates its cell state. We use ${\text{LSTM}}_{{{\text{enc}}}}$ to refer to the encoder LSTM cell, and ${\text{LSTM}}_{{{\text{dec}}}}$ to indicate the decoder LSTM cell.

LSTM-AE is specialized models tailored for time series data. They are highly effective in processing sequence data, particularly in domains like natural language processing and time series prediction. They excel at capturing temporal context. However, their complex structure may lead to longer training and inference times, and they might not perform optimally with limited data availability.

## Results

### Dataset

We propose a method for constructing seismic data from train vibration data. In this study, we used only the training vibration data to train the deep-learning model. However, to evaluate the performance of the model, seismic data that could potentially be measured on a train were required. We synthesized seismic data by incorporating a portion of the training data.

Initially, we divided the 100 train acceleration sensor datasets into three groups in the 80:10:10 ratio for training, validation, and testing purposes, respectively. The training data consisted exclusively of vibration data, which were used to train the deep learning model. Similarly, the validation data also comprised the training vibration data, aiding threshold determination for future earthquake detection. Finally, test data were employed to evaluate the deep learning model, which consisted of training vibration data and artificially generated seismic data derived from the training vibration data. The objective of this test was to assess the ability of the deep learning model to accurately detect earthquakes.

To synthesize the combined train vibration and earthquake data, we paired each individual train data with earthquake data of varying magnitudes, encompassing all training data and a range of earthquake data. For instance, considering earthquake data with the peak ground acceleration (PGA) value of 2.0 or higher, which comprised nine data points, and selected 10 training data points for the test set, we generated 90 data points. Because the training data sequences were quite long, we aimed to incorporate diverse segments while reducing their lengths. Therefore, we divided each training data sequence into five random segments and combined the earthquake data with each segment. Consequently, abnormal test data generation was based on multiplying the number of training data points by the number of earthquake data points and then multiplying by five. We obtained 450 abnormal test data points with a PGA values of 2.0 or higher. Additionally, from these abnormal test data points, we used only the training vibration data, excluding earthquake data, to construct the test normal data. Hence, normal and abnormal test data were identical.

To ensure consistency in the positioning of PGA during the superimposition of earthquake data and minimize the resulting variability, we consistently placed the PGA at the 3000th time point of the segmented train vibration data. Data containing only train vibrations without any superimposed earthquake data were used as the normal test data.

To reduce the noise in the train vibration data, we applied a bandpass filter with a frequency range of 1–45 Hz. This signal processing technique helped eliminate irrelevant frequencies and noise, and improved the performance of the deep learning model.

Furthermore, we employed robust normalization to mitigate the impact of outliers on the performance of our model. This normalization method utilizes robust statistical measures such as the median and interquartile range to scale the data and increase the resilience of the model against outliers.

The incorporation of a bandpass filter and robust normalization methods significantly enhanced the performance of our deep learning model for earthquake detection. The bandpass filter effectively removed noise from the train vibration data, enhancing discrimination between earthquake signals and normal vibrations. Meanwhile, the robust normalization technique reduces the influence of outliers, leading to more accurate and reliable results from our deep learning-based earthquake detection model.

### Experiment

We present an unsupervised deep-learning approach for earthquake detection on trains using anomaly detection. The model was trained on the training vibration data using unsupervised learning techniques. Specifically, we used a deep autoencoder to capture the underlying patterns within the training vibration data without relying on earthquake labels. Through this training process, the model learns to identify deviations from the learned patterns as anomalies that indicate potential earthquakes. During testing, the model was applied to new, unseen data from the train, and detected anomalies were flagged as potential earthquakes. This unsupervised approach eliminates the need for labeled earthquake data during training, making it valuable in scenarios were acquiring labeled data, particularly on trains, is challenging. Our experimental results demonstrate that the proposed approach achieves improved accuracy in detecting earthquakes on trains, even for small magnitudes.

For all deep-learning models, we employed the ADAM optimizer with a learning rate of 1e−3 and implemented it using Keras and TensorFlow.

The training process for all models utilized a mini-batch size of 32 and was conducted for 300 epochs (with the early stopping patience set to 5) using an NVIDIA GeForce RTX 3090 GPU, an Intel i7-8700K CPU, and 16 GB of RAM.

Finally, to determine whether the data were abnormal or normal, we employed the residuals between the test data and data generated by the trained model. The residual was subsequently utilized as an anomaly score; if the score surpassed a specific threshold, the data were classified as anomalous. To establish the threshold, we computed quantiles based on the validation data, comprising exclusively of normal data. Each quantile is used to define the corresponding threshold.

### Metrics

In our evaluation, we computed the values of the true positives (TP), false positives (FP), true negatives (TN), and false negatives (FN). TP represents the number of data points correctly classified as earthquake events. FP signifies the number of data points that are incorrectly classified as earthquake events when they are normal. FN denotes the number of normal KTX data points that experienced an earthquake, and TN denotes the number of KTX data points that did not experience an earthquake.

To assess the performance of our earthquake detection model, we utilized false alarm rate (FAR), missing alarm rate (MAR), and F1-score as evaluation metrics. FAR and MAR quantifies the proportion of incorrect predictions in the positive and negative class, respectively. The F1-score was calculated as the harmonic mean of precision and recall. The precision refers to the proportion of accurate predictions, whereas the recall represents the proportion of instances that were correctly predicted.

In the context of train earthquake detection, the terms "positive" and "negative" refer to the anomalous and normal states, respectively. An anomalous state indicates the presence of earthquake detection on the train, whereas a normal state denotes the absence of such detection.

This study employed unsupervised anomaly-detection techniques using a deep learning model capable of computing an anomaly score. The anomaly score quantified the level of anomalies based on the residuals between the generated and input values of the model. To distinguish between the normal and anomalous states, a separate criterion was established as a decision threshold. In this approach, a random quantile value from the validation data within the normal dataset is selected as the criterion. An anomaly score surpassing this threshold was classified as anomalous, whereas a score below the threshold was considered normal.

### Experimental results

In our evaluation, we conducted a comparative analysis of deep learning using unsupervised learning, the traditional STA/LTA and Recursive STA/LTA techniques for earthquake detection in trains. The data were divided based on different PGA conditions to assess the detection performance. Seven experiments were conducted for each model with the entire range of PGA values divided into seven bins, allowing us to assess the detection capability up to a specific PGA level. The F1 score, MAR, and FAR were selected as evaluation metrics to measure the overall performance of each model. The metric score of traditional STA/LTA and Recursive STA/LTA methods are summarized in Tables 1 and 2, in addition, the matric score of autoencoder, Conv-AE and LSTM-AE are summarized in Tables 3, 4 and 5 each. The PGA in the experimental resluts are described with unit of g(9.8m/s^2^)

**Table 1:** Metric score of STA/LTA model.

Threshold	Metric	PGA value (Unit: G)					
		≥ 0.01	≥ 0.03	≥ 0.05	≥ 0.07	≥ 0.1	≥ 0.15
2σ	FAR	0.978					
	MAR	0.000	0.000	0.000	0.000	0.000	0.000
	F1	0.672	0.674	0.670	0.668	0.670	0.669
3σ	FAR	0.356					
	MAR	0.122	0.093	0.058	0.024	0.000	0.000
	F1	0.786	0.808	0.819	0.849	0.849	0.843
4σ	FAR	0.209					
	MAR	0.233	0.144	0.089	0.047	0.000	0.000
	F1	0.776	0.830	0.857	0.887	0.902	0.891

**Table 2:** Metric score of recursive STA/LTA model.

Threshold	Metric	PGA value (Unit: G)					
		≥ 0.01	≥ 0.03	≥ 0.05	≥ 0.07	≥ 0.1	≥ 0.15
2σ	FAR	0.998					
	MAR	0.000	0.000	0.000	0.0000	0.000	0.000
	F1	0.667	0.668	0.667	0.668	0.667	0.667
3σ	FAR	0.454					
	MAR	0.089	0.062	0.036	0.022	0.000	0.000
	F1	0.769	0.794	0.796	0.804	0.815	0.809
4σ	FAR	0.243					
	MAR	0.178	0.109	0.064	0.029	0.000	0.000
	F1	0.801	0.835	0.861	0.883	0.888	0.885

**Table 3:** Metric score of autoencoder.

Threshold	Metric	PGA value (Unit: G)					
		≥ 0.01	≥ 0.03	≥ 0.05	≥ 0.07	≥ 0.1	≥ 0.15
2σ	FAR	0.307					
	MAR	0.127	0.031	0.000	0.000	0.000	0.000
	F1	0.801	0.819	0.850	0.845	0.843	0.837
3σ	FAR	0.044					
	MAR	0.553	0.364	0.176	0.096	0.000	0.000
	F1	0.599	0.745	0.877	0.911	0.969	0.961
4σ	FAR	0.027					
	MAR	0.716	0.593	0.344	0.178	0.067	0.000
	F1	0.434	0.565	0.778	0.881	0.951	0.983

**Table 4:** Metric score of Conv-AE.

Threshold	Metric	PGA value (Unit: G)					
		≥ 0.01	≥ 0.03	≥ 0.05	≥ 0.07	≥ 0.1	≥ 0.15
2σ	FAR	0.298					
	MAR	0.113	0.042	0.031	0.009	0.000	0.000
	F1	0.812	0.856	0.869	0.876	0.879	0.879
3σ	FAR	0.047					
	MAR	0.531	0.333	0.136	0.027	0.000	0.000
	F1	0.619	0.767	0.902	0.943	0.966	0.958
4σ	FAR	0.027					
	MAR	0.669	0.607	0.358	0.151	0.000	0.000
	F1	0.488	0.552	0.771	0.895	0.987	0.987

**Table 5:** Metric score of LSTM-AE.

Threshold	Metric	PGA value (Unit: G)					
		≥ 0.01	≥ 0.03	≥ 0.05	≥ 0.07	≥ 0.1	≥ 0.15
2σ	FAR	0.178					
	MAR	0.136	0.056	0.033	0.013	0.000	0.000
	F1	0.847	0.869	0.881	0.894	0.900	0.903
3σ	FAR	0.047					
	MAR	0.564	0.396	0.191	0.100	0.000	0.000
	F1	0.588	0.723	0.865	0.910	0.971	0.957
4σ	FAR	0.022					
	MAR	0.689	0.596	0.340	0.184	0.062	0.000
	F1	0.467	0.558	0.779	0.873	0.952	0.979

To implement and conduct experiments using the STA/LTA methods, we utilized the ObsPy library. STA/LTA values were determined using a grid search process to determine the optimal durations of the short- and long-term windows. The optimized values for STA and LTA were set to 4 and 400, respectively.

For deep learning using unsupervised learning, we employed several autoencoder-based models, including an autoencoder and convolutional autoencoder. To determine the appropriate threshold for distinguishing between anomalous and normal data, standard deviation is calculated using the validation data. The standard deviation serves as a critical metric, quantifying the extent to which data values deviate from the mean. Sigma(σ) represents the unit of measurement for the standard deviation. To distinguish between normal and anomalous data, we established a threshold based on the Sigma(σ) observed in the validation dataset, which exclusively contained normal data.

Approximately 95% of data points are situated within the range of 2σ from the mean, approximately 99.7% of data points are found within the range of 3σ from the mean, and the majority of data points are within 4σ from the mean. Since we calculated Sigma(σ) based on normal data as our reference, values that exceed these thresholds can be regarded as anomalies from a statistical perspective. This threshold ranged from 2σ to 4σ. Subsequently, we performed a systematic comparison to determine an optimal threshold for anomaly detection

**Figure 9:**
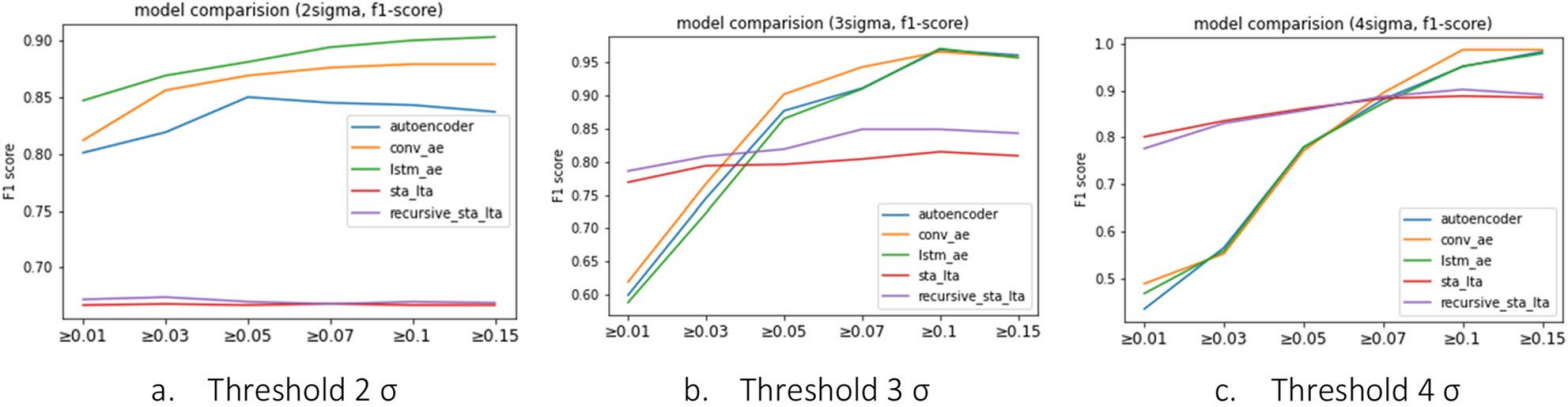
Metric of models (F1 score).

As depicted in Fig. 9, the results of our evaluation indicate that for the 3σ threshold, all deep learning models outperformed STA/LTA methods in the PGA range of 0.05 and above. Similarly, for the 4σ threshold, all deep learning models, except for the autoencoder model, exhibited better performance than STA/LTA methods. At the 2σ threshold, all deep learning models outperformed STA/LTA methods across all PGA ranges. In comparison to the conventional STA/LTA method, Recursive STA/LTA demonstrates similar overall performance; however, it notably outperforms the traditional STA/LTA approach at Threshold 3.

**Figure 10:**
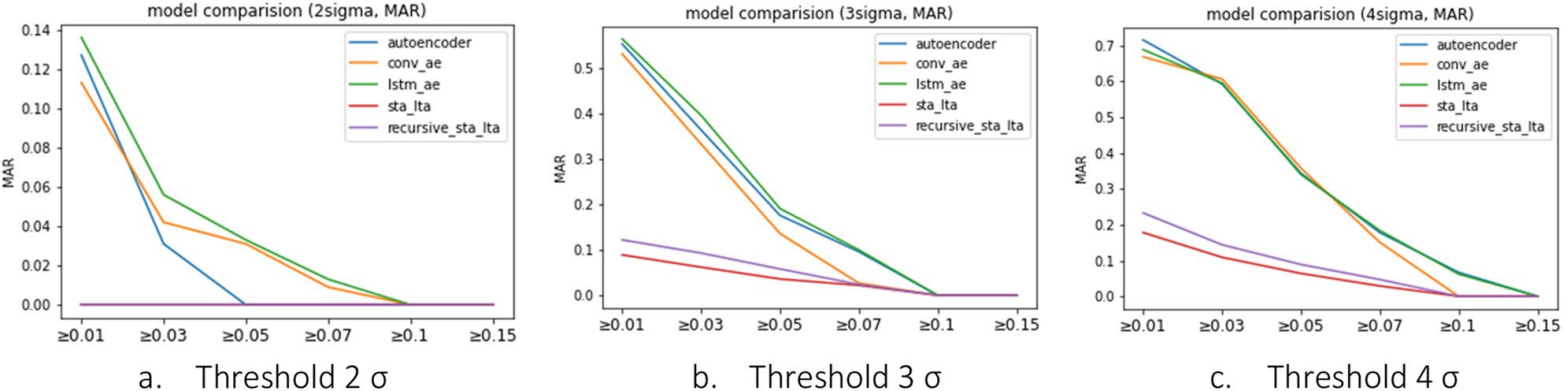
Metric of models (MAR).

**Figure 11:**
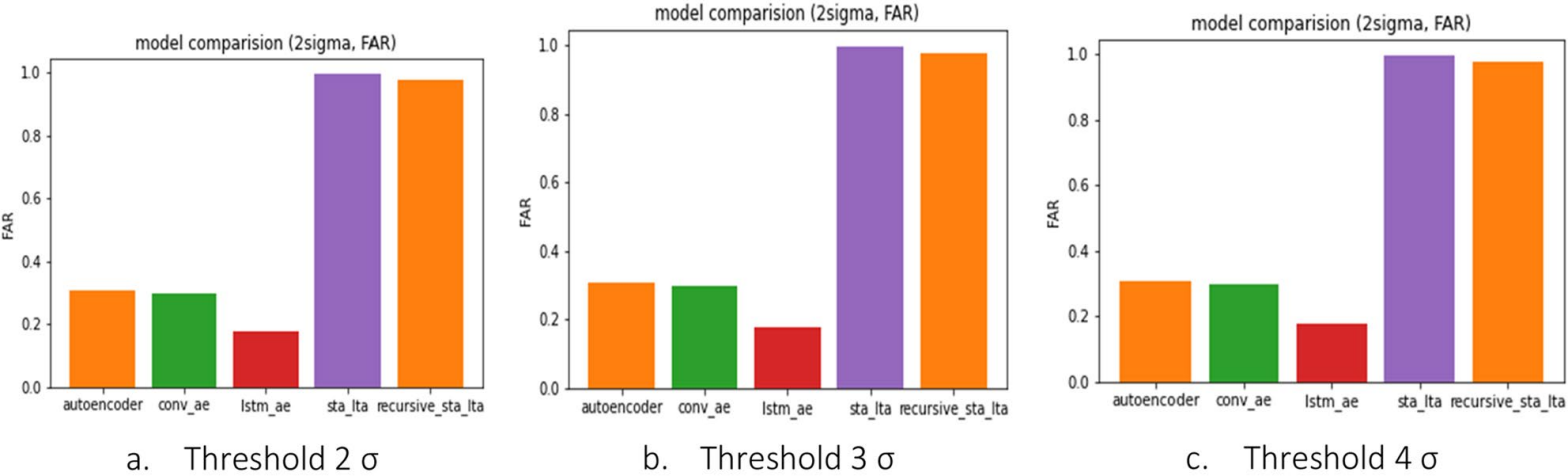
Metric of models (FAR).

However, as depicted in Fig. 10, STA/LTA methods consistently shows lower MAR values than deep learning models across all PGA ranges, as it tends to have a higher bias towards earthquake detection. Although this suggests a better MAR performance, the Fig. 11 shows FAR performance degrades profusely. Deep learning models demonstrated values close to 0 for FAR, indicating minimal instances of incorrectly predicting train vibrations as earthquakes, except at the 2σ threshold. In contrast, STA/LTA methods showed a relatively high FAR value, suggesting a tendency to perceive many situations as earthquakes. FAR performance of the conventional STA/LTA method is comparable to that of Recursive STA/LTA, albeit with a slightly superior FAR performance demonstrated by Recursive STA/LTA.

The findings indicate that deep learning models outperform STA/LTA methods in the PGA range of 0.05 and above for the 3σ threshold. For the 4σ threshold, all deep learning models, except the convolutional autoencoder, exhibited better performance than STA/LTA methods. Moreover, the deep learning models demonstrated significantly lower FAR values, indicating a minimal false alarm rate compared with STA/LTA methods. Therefore, for the effective detection of train vibrations and earthquakes, deep learning models show superior performance in terms of FAR, while demonstrating MAR results compffarable to those of STA/LTA methods, particularly for PGAs of 0.7 and above.

## Discussion

In this study, we utilized both the traditional STA/LTA and Rcursive STA/LTA technique and autoencoder-based models to distinguish between steady-state trains and abnormal-state seismic vibrations. By varying the threshold from 2σ to 4σ, we observed that different thresholds yielded better performances depending on the PGA range. Thus, if a specific PGA range requires a more definitive detection, adjusting the threshold can effectively capture that range.

Regarding the STA/LTA methods, at 2σ, the FAR was about 1.0, and the MAR was 0, indicating that all cases were determined to be earthquake events, rendering the model incapable of detecting earthquakes, as shown in Table 1. As the sigma(σ) value increased, the model performance improved partially, but the FAR remained at approximately 0.2, indicating a high probability of false detection of normal states, such as earthquakes.

In contrast, the autoencoder-based models showed a high MAR value for seismic accelerations below 0.07 g, and this tendency was more pronounced as the sigma(σ) value increased, as shown in Tables 3, 4 and 5. The F1-score, which evaluates the performance of the anomaly detection model, is lower than that of the STA/LTA methods. However, according to Korea's Railroad Safety Management Enforcement Regulations (2014)^13^, all train vehicles must be notified immediately when an earthquake with a magnitude of 0.067 g or higher occurs, because the risk of train derailment is significant for earthquakes of approximately 0.07 g magnitude. The results confirm that AE-based models perform better than the STA/LTA methods in detecting earthquakes that can cause train derailment.

The performances of the autoencoder-based models were analyzed to determine the most reasonable earthquake detection algorithm. All autoencoder-based models showed improved performance as the earthquake magnitude increased because distinguishing between train and earthquake vibrations became easier at larger magnitudes.

After analyzing the performance of the individual models based on earthquake size and threshold, it was determined that the Conv-Autoencoder model with a threshold of 4σ exhibited the highest performance for earthquakes of 0.15 g or greater. In this case, the F1 score was 0.987, the FAR was 2.7%, and the MAR was 0%, indicating the successful detection of all earthquakes greater than 0.15 g and very low false detection of normal conditions as earthquakes. However, this model is not suitable for detecting all earthquakes affecting train operations because the MAR exceeds 15% for seismic vibrations of 0.07 g or greater.

For earthquakes of 0.07 g or greater affecting train operation, the highest performance was achieved with the Conv-Autoencoder model with a threshold of 3σ. In this case, the F1 score was 0.943 and the FAR and MAR values were below 5%. Furthermore, the MAR was also 0% for earthquakes of 0.15 g or greater, showing comparable performance to the 4σ threshold of the Conv-Autoencoder model, which achieved the highest safety in detecting train earthquakes. However, with a threshold of four, the MAR value for all autoencoder-based models exceeded 15%, indicating a significant number of cases in which earthquake occurrences could not be detected. Based on a series of analyses, we determined that the Conv autoencoder model with a threshold of 3σ was the most reasonable algorithm for detecting earthquakes affecting train operations.

Among deep learning models with an Autoencoder (AE) architecture, Convolutional Autoencoders (Conv-AE) and Long Short-Term Memory Autoencoders (LSTM-AE) offer a distinct advantage by effectively capturing temporal contextual features, which traditional AEs may lack. As a result, Conv-AE and LSTM-AE generally outperform traditional AEs, particularly in the context of time series data analysis. This performance difference is notably evident when considering the F1-score, a comprehensive metric for assessing the performance of deep learning models.

At the 2σ threshold, Conv-AE and traditional AE demonstrate similar performance levels. However, as we move to the 3σ and 4σ thresholds, Conv-AE and LSTM-AE consistently outperform traditional AE. It is important to note that for PGA values above 1.0, the distinction among various deep learning models becomes less significant, with no substantial variation in results. Even at the 2σ threshold, while the performance gap between Conv-AE and traditional AE is relatively small, LSTM-AE exhibits a clear advantage over traditional AE. This trend underscores the enhanced ability of Conv-AE and LSTM-AE in capturing temporal contextual features when compared to traditional AE.

Our experiments demonstrated the capability of unsupervised learning to detect earthquakes, making it possible to build models relying solely on normal data even in situations where earthquake data are scarce or unavailable. Current earthquake early warning systems in train vehicles involve earthquake detection in structures, judgment in control centers, and notification of trains, all of which require considerable time to stop the train vehicle. In addition, it can only detect earthquakes at structures where seismometers are installed, therefore, it has the limitation of not being able to notify an immediate stop when an earthquake occurs on a line where a seismometer is not installed. The technology developed in this study has the advantage that all running trains can serve as seismographs, and that trains that detect earthquakes can immediately stop and provide earthquake warnings to following trains. Considering that high-speed trains can travel approximately 100 m/s at 300 km/h and take approximately 3.3 km to stop completely after braking is initiated, immediate train stopping is crucial for reducing the probability of derailment. The developed onboard earthquake detection and alarm technology offers the advantages of integrating existing procedures and enabling faster train stoppage after an earthquake occurs. Additionally, existing earthquake early warning systems have limitations as many sensors must be attached to the structure to cover the entire railway line. Developed technology can reduce the maintenance cost by installing sensors directly in the train, and it also has strengths in network quality.

Also, the seismic vibration used in this study has a limitation in that it is not the vibration measured on a moving train. However, feasibility of distinguishing normal-state train vibration from abnormal-state vibration such as earthquakes was confirmed, in addition, the size of earthquakes that can be distinguished within a train was analyzed. Therefore, it is expected that more advanced models, such as detecting earthquake primary wave in moving train, will be able to be developed in the future when this technology is commercialized and actual seismic vibration which can be measured in moving train vehicle is obtained.

As this study employs anomaly detection, it is conceivable that non-seismic noise sources such as strong winds or collisions could trigger detections. Nonetheless, our primary research focus was to assess the applicability of anomaly detection for earthquake identification, and our findings validated this. The detection of non-earthquake noises remains meaningful, as they can pose risks to train safety. Future investigations can expand on this by gathering additional data and enhancing the model.

## Data Availability

The datasets used and/or analyzed during the current study are available from the corresponding author on reasonable request. The data sources, collection methods, and processing techniques are summarized below. Data Sources and Collection: We utilized train vibration data from the South Korean KTX high-speed trains and seismic data observed by seismometers. Data Types: Both train vibration data and seismic data consist of time-series datasets with values that vary over time. Data Processing and Analysis: For the creation of training data, we exclusively employed train vibration data. Evaluation data were synthesized by combining train vibration data with seismic data. Standardization preprocessing was applied to all data based on the training dataset. Subsequently, seismic detection outcomes were predicted using the STA/LTA model and three deep learning models. Data Repository: The data was stored in a local repository within our research laboratory for the duration of the study. Data Access and Usage Conditions: Access to the data and the terms of its utilization are outlined as follows. The data's accessibility for use, whether open to everyone or subject to limitations, is specified. Contact Information: For inquiries, please contact mintaekyoo@gachon.ac.kr.
